# Transcriptomic Analysis of Green Leaf Plants and White–Green Leaf Mutants in *Haworthia cooperi* var. *pilifera*

**DOI:** 10.3390/genes15050608

**Published:** 2024-05-10

**Authors:** Peiling Li, Maofei Ren, Juanjuan Chen, Jianhua Yue, Songhu Liu, Qingsong Zhu, Zhiyong Wang

**Affiliations:** College of Horticulture, Xinyang Agriculture and Forestry University, Xinyang 464000, China; lxmlpl@163.com (P.L.); chenjj91@163.com (J.C.); jhyues@163.com (J.Y.); lsh711906@163.com (S.L.); zqs43@126.com (Q.Z.); wzhygansu@163.com (Z.W.)

**Keywords:** white–green leaf mutant, *Haworthia cooperi* var. *pilifera*, transcriptome analysis, leaf color

## Abstract

*Haworthia cooperi* var. *pilifera* is a succulent plant with ornamental value. The white–green leaf mutant (*wl*) showed a significant difference in leaf color from the wild-type plant (WT). In this study, we integrated the transcriptomes of *wl* and WT plants to screen differentially expressed genes related to leaf color variation. The results of transcriptome analysis showed that 84,163 unigenes were obtained after de novo assembly and the NR database annotated the largest number of unigenes, which accounted for 57.13%, followed by NT (43.02%), GO (39.84%), Swiss-Prot (39.25%), KEGG (36.06%), and COG (24.88%). Our finding showed that 2586 genes were differentially expressed in the two samples, including 1996 down-regulated genes and 590 up-regulated genes. GO analysis predicted that these differentially expressed genes (DEGs) participate in 12 cellular components, 20 biological processes, and 13 molecular function terms and KEGG analysis showed that metabolic pathways, plant–pathogen interaction, glycerophospholipid metabolism, endocytosis, plant hormone signal transduction, and ether lipid metabolism were enriched among all identified pathways. Through functional enrichment analysis of DEGs, we found that they were involved in chloroplast division and the biosynthesis of plant pigments, including chlorophyll, carotenoids, anthocyanin, and transcription factor families, which might be related to the formation mechanism of leaf color. Taken together, these results present insights into the difference in gene expression characteristics in leaves between WT and *wl* mutants and provide a new insight for breeding colorful leaf phenotypes in succulent plants.

## 1. Introduction

*Haworthia*, which is a succulent plant having transparent leaves, is widely grown and valued commercially as ornamentals [1,2]. In the past few decades, researchers have focused on tissue culture [3], new phenolics separation [4], propagation methods [5], DNA fingerprinting, and the botanical study [6] of plants from *Haworthia* species. Genomic information about this species is not yet available. *Haworthia cooperi* var. *pilifera*, a succulent plant from South Africa, is quite popular in China and belongs to the genus *Haworthia* and the family Liliaceae. Previously, during the breeding of *H. cooperi* var. *pilifera*, a white–green leaf mutant (*wl*) was obtained. The *wl* mutant displayed a distinct white–green phenotype, which is quite different from the wild-type plant (WT) with a green leaf and it is a very valuable resource because a white–green leaf is one of the most special traits in succulent plants.

Leaf color is a significant ornamental trait of plants and the formation mechanism of leaf color is very complex [7,8,9,10,11,12]. The color of plant leaves is mainly determined by the categories and content of pigments, such as chlorophyll, carotenoids, and anthocyanins [13,14]. Previous studies found that the molecular mechanism of plant leaf color involves many different genes [15,16]. Genes for chloroplast development [17] and division [18], chlorophyll biosynthesis [19], carotenoid biosynthesis, and anthocyanin synthesis [20,21,22] have been identified in many plants, as well as transcription factors [23,24,25] associated with leaf color. 

Transcriptome sequencing is a new technique to study the expression level of genes, which contributes to molecular biology research in both model and nonmodel plants and is not dependent on an existing genomic sequence. Previous transcriptome sequencing analyses related to leaf color have focused on specific plants, such as birch (*Betula platyphylla × B. pendula*) [26], tea (*Camellia sinensis* (L.) O. Kuntze) [27], and *Acer palmatum* Thunb. (maple) [28]. Transcriptome analysis of green leaves and red–purple leaves from *Fraxinus angustifolia* showed that there were nine differentially expressed genes related to anthocyanins [29]. However, research on the change in leaf color has been limited, especially in succulent plants.

In this study, transcriptome sequencing analysis was performed on the leaves of the white–green leaf mutant (*wl*) and green leaf plant (WT) of *H. cooperi* var. *pilifera* to characterize the gene expression and then to screen out the DEGs that may be related to the metabolic process of leaf color expression. The results provide a theoretical basis for elucidating the mechanism of leaf color formation, which can be used in succulent plant breeding to obtain better ornamental phenotype accessions.

## 2. Materials and Methods

### 2.1. Plant Materials

*H. cooperi* var. *pilifera*, a popular succulent cultivar, was used in this experiment. The seedlings with green leaf (WT)and white–green leaf (*wl*) were grown on MS medium with a thermoperiod of 25 °C (day) and 22 °C (night) and a photoperiod of 16 h. The leaves from healthy plants were harvested and stored at −80 °C for subsequent RNA-Seq. Three biological replicates were performed for each group.

### 2.2. RNA Extraction, mRNA-seq Library Construction, and Sequencing

The total RNA of the two groups (WT and *wl*) was extracted using a Plant RNA Kit (TaKaRa, Tokyo, Japan) following the manufacturer’s instructions. The quality and quantity of the total RNA were assessed at absorbance ratios of OD_260/280_ and OD_260/230_ and with 1% agarose gel electrophoresis. The replicates were mixed to produce 2 pooled samples for the sequencing analysis. After mRNA-seq libraries were generated using the VAHTS mRNA-seq v2 Library Prep Kit for Illumina (Vazyme, NR601, Nanjing, China) following the manufacturer’s recommendations and the library concentration was measured using the Qubit^®^ RNA Assay Kit in Qubit^®^ 3.0 for preliminary quantification. The insert size was assessed using the Agilent Bioanalysis 2100 system and found to be consistent with expectations; then, the qualified insert size was accurately quantified using qPCR with the StepOnePlus Real-Time PCR system (ABI, Norwalk, CT, USA). The clustering of the index-coded samples was performed on a cBot Cluster Generation System (Illumina, San Diego, CA, USA) according to the manufacturer’s instructions. After cluster generation, the libraries were sequenced on an Illumina HiSeq X Ten platform with the 150 bp paired-end module.

### 2.3. Sequence Analysis, De Novo Assembly, and Annotation

The raw reads were cleaned by removing adaptor sequences, low-quality sequences (reads with ambiguous bases ‘N’), and reads with Q-value < 20 bases. Then, we discarded reads with lengths less than 150 bp. Next, the remaining high-quality reads were de novo assembled into transcripts using the Trinity pipeline with the default settings. Then, the contigs were connected into transcript sequences to recover full-length transcripts across a broad range of expression levels, with the redundant duplicated reads removed. The longest transcript from the potential assembled alternative splicing transcript components was selected as the unigene of each set for subsequent analysis.

The unigenes were compared with public databases and functional annotations were made based on similarity. The data used for comparison included the NCBI nonredundant proteins (NCBI NR), Gene Ontology (GO), Clusters of Orthologous Genes (COG), and Kyoto Encyclopedia of Genes and Genomes (KEGG) databases.

### 2.4. Identification of Differentially Expressed Genes (DEGs)

The DEGs in the WT vs. *wl* comparison were defined using an FDR (false discovery rate) of ≤0.05 and an absolute value of log2 abundance ratio of ≥1 (twofold change). Then, the identified DEGs were subjected to GO functional analysis and KEGG pathway analysis.

## 3. Results

### 3.1. Sequencing and De Novo Assembly

The main characteristics of the two RNA-seq libraries are summarized in Table 1. The number of raw reads per library ranged from ~42.1 to ~47.1 million. After filtering low-quality reads containing adaptors and low-quality sequences (defined as containing <90% confidently identified bases), we obtained 40,952,422 and 45,235,250 clean reads, which corresponded to 6,142,863,400 and 6,785,287,500 base pairs, respectively. The proportion of clean reads from each library was >96.04%. The transcriptomes of both the WT and *wl* samples were represented by at least four million clean reads each, which allowed for quantitative analysis of gene transcription. The raw sequencing data were submitted to the NCBI Sequence Read Archive (SRA) database with accession number SUB8069563.

**Figure 1:**
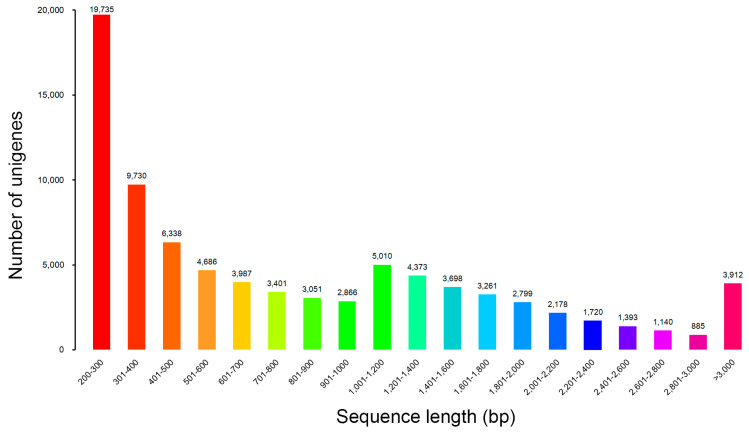
Length distribution of unigenes. The *x*-axis indicates sequence lengths from 1 to 20,000 nt. The *y*-axis indicates the corresponding number of unigenes.

Through de novo assembly, 84,163 unigenes with a mean length of 1013 bp were obtained. The length of the assembled unigenes ranged from 200 to 15,563 bp with an N50 length of 1668 bp (Table S1). The sequence length distribution of these unigenes is shown in Figure 1. Most unigenes were from 200–300 bp, while the longest unigene was 15,563 bp.

### 3.2. Gene Annotation and Functional Calculation

Alignment between the unigenes and public databases such as NR, NT, Swiss-Prot, KEGG, Clusters of Orthologous Genes (COG), and GO was performed and the results with the best alignment were used to determine the sequence direction of each unigene. The number and percentage of unigenes aligned to each database are shown in Table S2. A total of 48,085 (57.13%), 36,210 (43.02%), 33,036 (39.25%), 30,353 (36.06%), 20,941 (24.88%), and 33,527 (39.84%) unigenes had significant hits in NR, NT, Swiss-Prot, KEGG, COG, and GO, respectively.

**Figure 2:**
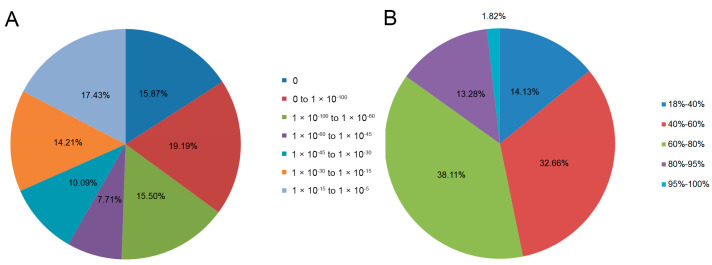
Homology search results for query sequences aligned by BLASTx to the NR database. (**A**) E-value distribution of unigene BLASTx hits in the NR database with an E-value threshold of 1.0 × 10^−5^. (**B**) Identity distribution of the top BLAST hits for each unigene.

**Figure 3:**
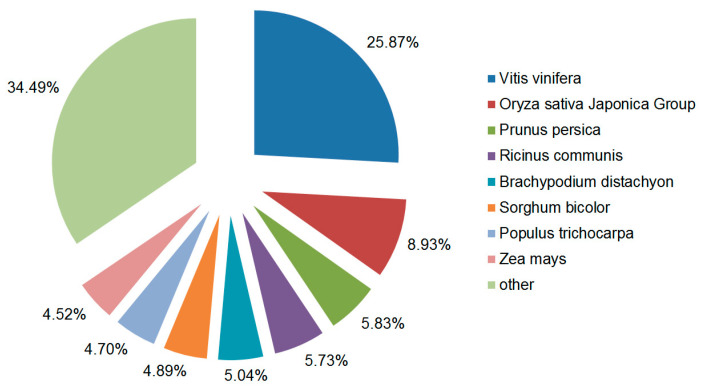
Percentage of the *H. cooperi* var. *pilifera* unigenes showing similarity to genes in other plant species. The *H. cooperi* var. *pilifera* unigenes were aligned by BLASTx to the NR database.

For the E-value distribution, 58.26% of the unigenes had confidence levels of at least 1 × 10^−45^ (Figure 2A). A total of 53.21% had alignment identities greater than 60% with existing proteins in the NR database (Figure 2B). To gain insight into the percentage of the 47,851 unigenes that showed similarity to genes in other plant species, we analyzed the species distribution of Haworthia cooperi var. pilifera in the NR database, in which 65.51% of the genes were annotated as having the highest similarity to genes from 8 species, the largest number among the databases examined (Figure 3). Among the various plants, the 12,378 unigenes had the highest number of unigenes with the greatest similarity to Vitis vinifera (25.87%), 8.93% to Oryza sativa Japonica Group, 5.83% to Prunuis persca, 5.73% to Ricinus communis, and 5.04% to Brachypodium distachyon.

**Figure 4:**
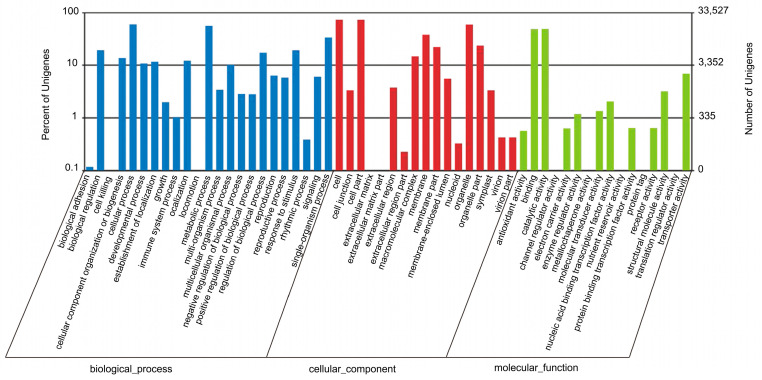
Distribution of Gene Ontology (GO) annotations of all unigenes from *H. cooperi* var. *pilifera*. A total of 33,527 unigenes were assigned GO terms and they were classified into three GO categories: biological process, cellular component, and molecular function. The left *y*-axis indicates the percentage of unigenes in a specific category. The right *y*-axis indicates the number of unigenes in a category.

The GO analysis allowed the functional classification of 33,527 unigenes. As shown in Figure 4, the unigenes could be categorized into 56 functional groups on the terms of sequence homology. GO terms are grouped into three categories: biological process, cellular component and molecular function. In the biological process category, the largest number of unigenes were associated with ‘cellular process’ (59.55%), followed by ‘metabolic process’ (55.79%) and ‘single-organism process’ (33.67%). On the other hand, only a few genes were associated with ‘biological adhesion’ (0.12%), ‘locomotion’ (0.10%), and ‘cell killing’ (0.02%). The major cellular component terms in the GO classification were ‘cell’, ‘cell part’ and ‘organelle’, while the predominant molecular function terms were ‘binding’ and ‘catalytic activity’.

**Figure 5:**
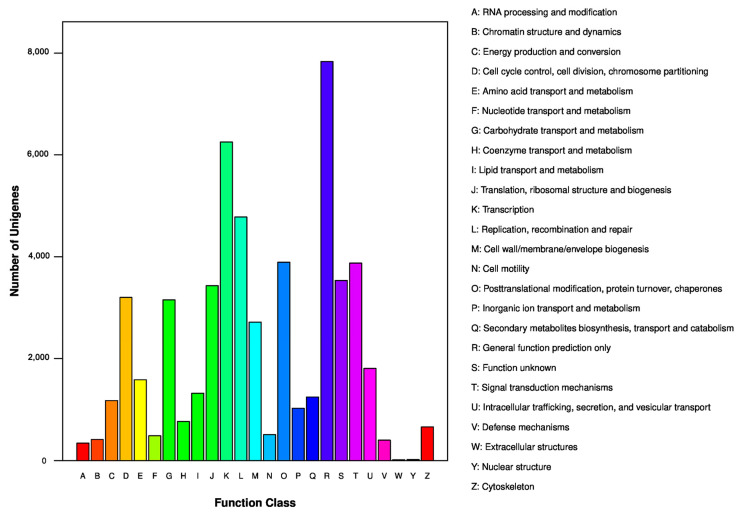
COG functional classifications of all unigenes from *H. cooperi* var. *pilifera*. A total of 20,941 sequences had a COG classification among 25 categories.

We further classified the functions of the unigenes by using the COG database. A total of 20,941 unigenes were assigned to 25 specific COG categories, which are listed in Figure 5. The largest group was ‘general function prediction only’ with 7834 unigenes (37.41%), followed by ‘transcription’ with 6253 unigenes (29.86%), ‘replication, recombination, and repair’ with 4778 unigenes (22.82%), ‘posttranslational modification, protein turnover, and chaperones’ with 3888 unigenes (18.57%), and ‘signal transduction mechanisms’ with 3872 unigenes (18.49%). ‘Function unknown’ contained 3529 unigenes (16.85%), ‘translation, ribosomal structure, and biogenesis’ contained 3428 unigenes (16.37%), ‘cell cycle control, cell division, and chromosome partitioning’ contained 3197 unigenes (15.27%), ‘carbohydrate transport and metabolism’ contained 3150 unigenes (15.04%), and ‘cell wall/membrane/envelope biogenesis’ contained 2712 unigenes (12.95%). The categories ‘nuclear structure’ and ‘extracellular structures’ represented the smallest groups.

**Figure 6:**
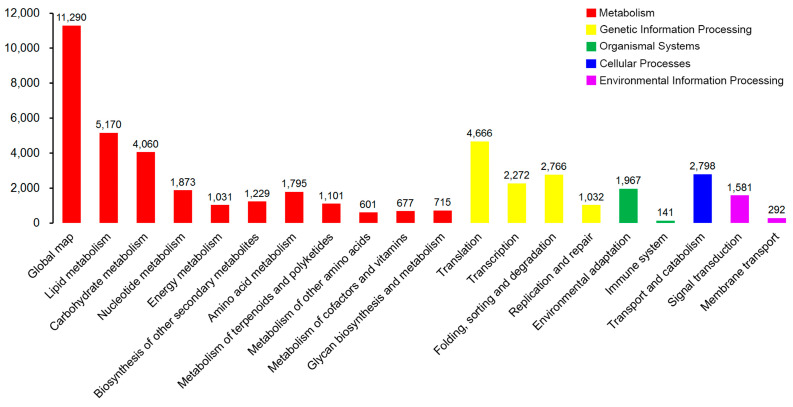
KEGG pathway assignments of the assembled unigenes. A total of 30,353 unigenes were mapped to 128 KEGG pathways belonging to five categories: metabolism (red), genetic information processing (yellow), organismal systems (green), cellular processes (blue), and environmental information processing (purple).

KEGG was used as a higher-order functional annotation to understand the metabolic and biological pathways and functions of the gene products in the cells. We annotated and mapped 128 KEGG pathways for 30,353 unigenes (Figure 6). These pathways were distributed in five main categories: metabolism (97.33%), genetic information processing (35.37%), organismal systems (6.94%), cellular processes (9.22%), and environmental information processing (6.17%). The KEGG pathway analysis showed that the unigenes were mainly located in metabolic pathways (ko01100, 8107 unigenes), biosynthesis of secondary metabolites (ko01110, 3183 unigenes), endocytosis (ko04144, 2079 unigenes), and glycerophospholipid metabolism (ko00564, 2059 unigenes).

### 3.3. Analysis of Potential DEGs

**Figure 7:**
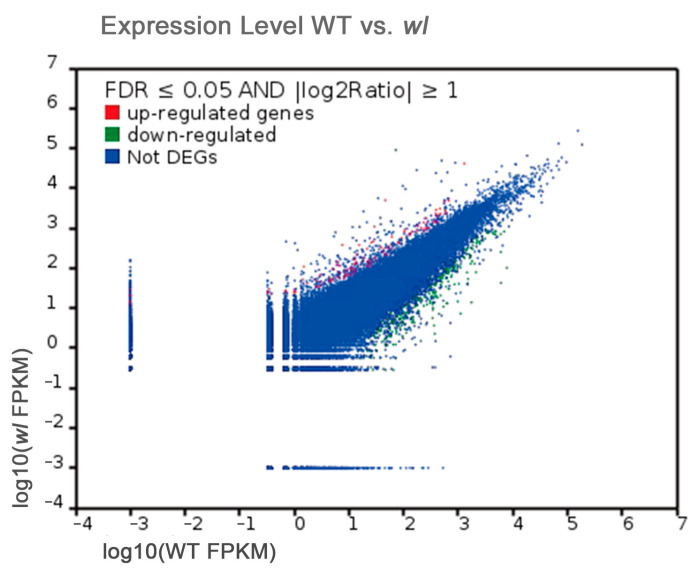
Gene expression levels between the two libraries (WT and *wl*). Red dots represent transcripts more prevalent in the *wl* library, green dots show those present at a lower frequency in *wl*, and blue dots indicate transcripts that did not change significantly.

Based on expression abundance (FPKM values), the gene expression levels of the two samples are shown in Figure 7. A total of 2586 genes were found to be differentially expressed in *wl* compared with WT using a threshold of ratio change ≥2 and false discovery rate (FDR) ≤0.05. A higher number of down-regulated than up-regulated genes was observed in the *wl* sample. In total, 590 DEGs showed up-regulated expression, while 1996 DEGs were down-regulated (Table S3).

### 3.4. GO and KEGG Enrichment Analyses of DEGs

**Figure 8:**
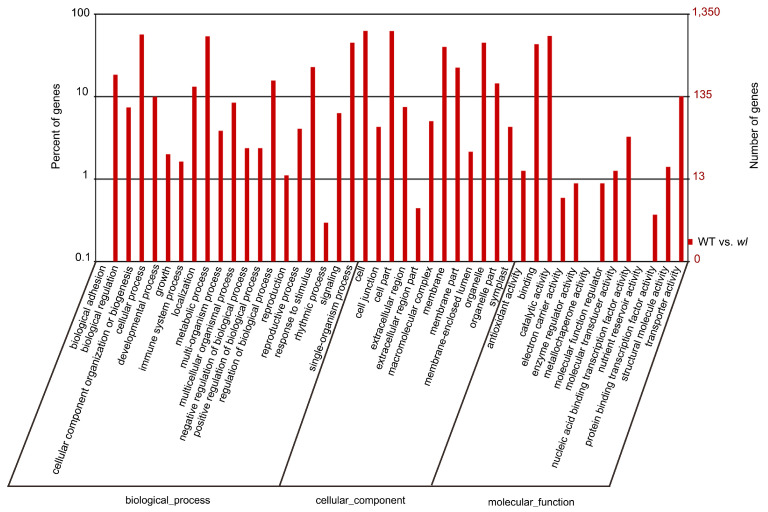
GO enrichment analysis of DEGs in WT vs. *wl*. Enrichment in the GO categories ‘biological process’, ‘cellular components’, and ‘molecular function’ categories is shown.

GO annotation was used to determine the functions of the DEGs. The DEGs were divided into 45 functional terms in three categories: biological process, cellular component, and molecular function (Figure 8). Cell (846 DEGs) was the most common term, followed by cell part (842), cellular process (764), catalytic activity (735), metabolic process (727), single-organism process (609), organelle (609), binding (584), and membrane (541). The GO terms in all three ontologies with the highest enrichment of DEGs are shown in Table 2.

**Figure 9:**
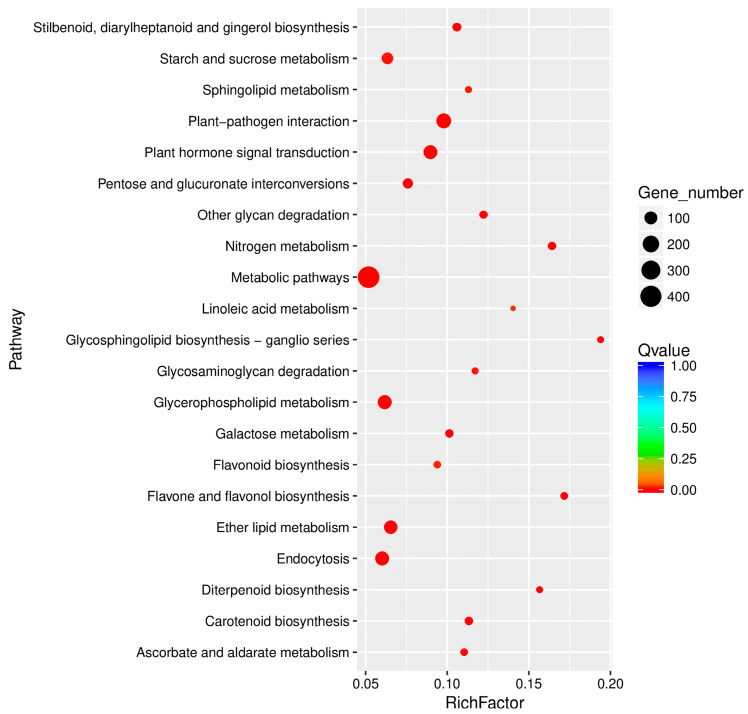
Top 20 KEGG pathways enriched in DEGs in WT vs. *wl*.

Further classifications under each category are also listed. To understand the biological functions of the DEGs, we also mapped these genes to pathways in the KEGG database. The DEGs were functionally assigned to 109 KEGG pathways and the 20 pathways most enriched in DEGs are shown in Figure 9. Among the mapped pathways, 24 pathways were significantly enriched (*p*-value ≤ 0.01) (Table S4). ‘Metabolic pathways’ was the most enriched pathway (420 DEGs), followed by ‘plant–pathogen interaction’ (149), ‘glycerophospholipid metabolism’ (127), ‘endocytosis’ (125), ‘plant hormone signal transduction’ (124), and ‘ether lipid metabolism’ (117).

## 4. Discussion

### 4.1. Identification of DEGs Involved in Chloroplast Division

Leaf-color mutants are affected by chloroplast development and division, which can directly or indirectly affect chloroplast structure and number and other metabolic processes performed by these complex macromolecular machines, which have components positioned on both the inner and outer envelope surfaces [18]. Many proteins, such as FtsZ1, tubulin-like protein, MinD, and ARTEMIS, are involved in chloroplast division [30,31,32,33,34,35]. In this process, tubulin-like proteins localize to a ring at the site of plastid constriction, which drives chloroplast division. In our study, we found that six unigenes of seven DEGs encoding tubulin proteins were down-regulated in the white–green leaf mutant, including Unigene5427_All, CL2099.Contig19_All, CL2099.Contig8_All, CL2099.Contig6_All, Unigene8230_All, and CL2099.Contig4_All (Table S5); one gene (Unigene1981_All) encoding an α-tubulin suppressor was up-regulated, suggesting that these genes might be associated with chloroplast division and might therefore affect leaf color.

### 4.2. Identification of DEGs Involved in Plant Pigment Synthesis

Plant leaf color is related to the types and contents of pigments in the cells. Enzymes are needed for the synthesis of plant pigments; for example, Mg-chelatase and protoporphyrinogen oxidase are involved in chlorophyll synthesis, zeta-carotene desaturase and zeaxanthin epoxidase in carotenoid synthesis, and chalcone synthase in anthocyanin synthesis.

In our work, one gene (CL11028.Contig1_All) encoding magnesium chelatase was significantly down-regulated, while Unigene17322_All, encoding protoporphyrinogen oxidase, was up-regulated, as was one gene encoding the probable chlorophyll (ide) b reductase NYC1. Magnesium chelatase is a heterotrimeric enzyme complex that catalyzes a key regulatory and enzymatic reaction in chlorophyll biosynthesis [36], protoporphyrinogen oxidase is a common enzyme in chlorophyll biosynthesis [20], and chlorophyll b reductase NYC1 and NOL (NYC-like) form the chlorophyll b reductase complex, which is necessary to catalyze the first step of chlorophyll b degradation [37].

Seven zeta-carotene desaturase genes (three up-regulated and four down-regulated), three zeaxanthin epoxidase genes (one up-regulated and two down-regulated), and one 15-cis-zeta-carotene isomerase gene (up-regulated) were differentially expressed. Zeta-carotene desaturase is a key enzyme that controls β-carotene production upstream of carotenoid biosynthesis [21]. Zeaxanthin epoxidase is an important enzyme that converts zeaxanthin to violaxanthin [38]. 15-Cis-zeta-carotene isomerase can isomerize 9,15,9′-tri-cis-zeta-carotene into 9,9′-di-cis-zeta-carotene, which is the last identified gene in the plant carotenoid biosynthetic pathway [39].

Five genes predicted to encode naringenin-chalcone synthase were down-regulated and Unigene26705_All was significantly decreased (|log2Ratio| > 10). Chalcone synthase is the first key enzyme and rate-limiting enzyme in flavonoid synthesis [40].

Four genes encoding flavonoid 3′-monooxygenase were down-regulated in the white–green leaf mutant, including CL3495.Contig2_All, Unigene632_All, Unigene20726_All, and CL3495.Contig1_All. Flavonoid 3′-monooxygenase is responsible for one reaction in flavonoid compound biosynthesis [41]. 

We identified 15 differentially expressed cytochrome P450 genes, of which 3 genes were up-regulated and 12 down-regulated. Cytochrome P450 is a monooxygenase encoded by a supergene family that is involved in the synthesis of many terpenoids in plants, including carotenoids [42]. In addition, we found six genes encoding chloroplast chlorophyll a/b-binding protein, all of which were down-regulated. Chlorophyll a/b-binding protein can enhance photosynthesis in the chloroplasts of plants [43].

### 4.3. Identification of DEGs Involved in Anthocyanin Transport

In our study, 22 genes encoding ABC (ATP binding cassette) transporter, 5 genes encoding MATE (multidrug and toxic compound extrusion), and 8 genes encoding GST (glutathione S-transferase) were found among the DEGs. All three of these transporter types can transport anthocyanin in plants by various pathways. In this work, four genes encoding ABC transporters were up-regulated in *wl*, while the others were down-regulated. The up-regulated genes were CL5908.Contig1_All, Unigene14496_All, CL1076.Contig2_All, and CL9731.Contig1_All, as shown in Table S5. Both the genes encoding MATE and those encoding GST were down-regulated in *wl* compared to WT. These results suggested that the transporters might transport plant pigments by complex molecular mechanisms.

### 4.4. Identification of DEGs Encoding Transcription Factors Involved in Leaf Color

Transcription factors are involved in chloroplast development through upstream regulation of gene expression pertaining to chlorophyll, anthocyanin, and carotenoid biosynthesis in plants, such as the MYB, bHLH, and WD40 transcription factor families. MYB transcription factors, which regulate the expression of key enzymes in the early flavonoid biosynthetic pathway, enhance the synthesis and accumulation of proanthocyanidins in plants [44]. In tobacco, overexpression of LeAN2, which encodes an anthocyanin-associated R2R3-MYB transcription factor, induced the expression of several anthocyanin biosynthetic genes and the content of anthocyanin was markedly higher in the transgenic tobacco than in wild-type plants [33]. Here, 13 MYB transcription factors were identified; 1 was up-regulated and the others were down-regulated. Recent studies have revealed that bHLH transcription factors also regulate chlorophyll biosynthesis. For example, PIF1 negatively regulates chlorophyll biosynthesis, while pif1 mutant seedlings accumulate excess free protochlorophyllide when grown in the dark, with consequent lethal bleaching upon exposure to light [14]. In this study, we found 10 bHLH family genes that were down-regulated in *wl*, as shown in Table S5. WD40 transcription factors regulate the synthesis and accumulation of anthocyanin by affecting the expression of anthocyanin structural genes [45]. TTG1, a WD-repeat (WDR) protein, acts as an important regulator of enzymes controlling proanthocyanidin biosynthesis in plants [25]. In the present study, five genes encoding WD40 were down-regulated, while one was up-regulated. All these results suggested that transcription factors might constitute a complex network regulating leaf color in plants.

## 5. Conclusions

Though the high-throughput transcriptome sequencing for the leaves of green leaf plant and white–green leaf plant from *H. cooperi* var. *pilifera*, we obtained 40,952,422 and 45,235,250 clean reads, which corresponded to 6,142,863,400 and 6,785,287,500 base pairs, respectively. In total, 84,163 unigenes with a mean length of 1013 bp were obtained after de novo assembly. A total of 48,085 (57.13%), 36,210 (43.02%), 33,036 (39.25%), 30,353 (36.06%), 20,941 (24.88%), and 33,527 (39.84%) unigenes were annotated in NR, NT, Swiss-Prot, KEGG, COG, and GO, respectively. There were 2586 DEGs between the two samples, with 590 up-regulated and 1996 down-regulated. Transcriptome data showed that DEGs had significant expression differences in the two samples, which are associated with chloroplast division and the biosynthesis of plant pigments, such as chlorophyll, carotenoids, anthocyanin, and transcription factor families. We predicted that they were the key structural genes and the main factors of the molecular regulation mechanism of the leaf color of *H. cooperi* var. *pilifera*.

## Figures and Tables

**Table 1:** The statistics of sequencing.

Summary	WT	*wl*
Total raw reads	42,199,192	47,096,827
Total clean reads	40,952,422	45,235,250
Total clean nucleotides (bp)	6,142,863,400	6,785,287,500
Q20 (%)	97.28	97.16
Q30 (%)	93.49	93.20
N content (%)	0.01	0.01

**Table 2:** The most reliable top 10 significantly enriched pathways of DEGs in the cellular component, molecular function, and biological process.

Type	Pathway	Number	*p*-Value
Cellular component	cell wall	80	7.34 × 10^−15^
	external encapsulating structure	80	8.57 × 10^−15^
	extracellular region	101	9.41 × 10^−11^
	intrinsic component of plasma membrane	56	3.46 × 10^−9^
	cell periphery	257	1.33 × 10^−8^
	integral component of plasma membrane	40	1.43 × 10^−5^
	plant-type cell wall	32	1.85 × 10^−5^
	plasma membrane part	58	2.77 × 10^−5^
	membrane	541	0.00807
	plant-type vacuole membrane	15	0.01089
Molecular function	symporter activity	27	1.35 × 10^−6^
	xyloglucan:xyloglucosyl transferase activity	12	2.57 × 10^−6^
	transmembrane transporter activity	124	1.40 × 10^−5^
	carboxylic acid transmembrane transporter activity	28	1.70 × 10^−5^
	amino acid transmembrane transporter activity	23	1.95 × 10^−5^
	organic acid transmembrane transporter activity	28	3.15 × 10^−5^
	superoxide dismutase activity	10	6.47 × 10^−5^
	oxidoreductase activity, acting on superoxide radicals as acceptor	10	6.47 × 10^−5^
	anion transmembrane transporter activity	46	0.00014
	ion transmembrane transporter activity	89	0.0002
Biological process	response to high light intensity	21	2.21 × 10^−5^
	amino acid transmembrane transport	23	2.89 × 10^−5^
	anion transport	53	4.50 × 10^−5^
	carbon utilization	7	7.62 × 10^−5^
	response to light intensity	25	9.11 × 10^−5^
	organic acid transmembrane transport	24	0.00029
	amino acid transport	23	0.00034
	response to reactive oxygen species	24	0.00055
	carbohydrate metabolic process	114	0.00061
	xyloglucan metabolic process	12	0.00067

## Data Availability

The original contributions presented in the study are included in the article/Supplementary Materials, further inquiries can be directed to the corresponding author.

## Supplementary Materials

The following supporting information can be downloaded at: https://www.mdpi.com/article/10.3390/genes15050608/s1, Table S1: Statistics of de novo assembly; Table S2: Annotation of unigenes with different public database; Table S3: DEGs of the two groups; Table S4: KEGG annotation of DEGs (*p* value≤ 0.01); Table S5: The differentially expressed genes related to plant pigment in leaf color mutant compared to control plant of *Haworthia cooperi* var. *pilifera.*
