# Supplementary material for: Transcriptomic Analysis of Green Leaf Plants and White–Green Leaf Mutants in Haworthia cooperi var. pilifera
Source: Genes (Basel). 2024 May 10;15(5):608. doi: 10.3390/genes15050608 (PMC11121492; doi:10.3390/genes15050608)
Supplement: Supplementary file 1 [file genes-15-00608-s001.zip › Table S1.pdf]

Table S1 Statistics of de novo assembly

	Total number	Total length (bp)	Mean length (bp)	Longest unigene (bp)	Shortest unigene (bp)	N50 (bp)
Unigenes	84,163	85,259,353	1,013	15,563	200	1,668
