# Supplementary material for: Transcriptomic Analysis of Green Leaf Plants and White–Green Leaf Mutants in Haworthia cooperi var. pilifera
Source: Genes (Basel). 2024 May 10;15(5):608. doi: 10.3390/genes15050608 (PMC11121492; doi:10.3390/genes15050608)
Supplement: Supplementary file 1 [file genes-15-00608-s001.zip › Table S2.pdf]

Table S2 Annotation of unigenes with different public database

Database	Number of unigenes	Percentage (%)
Annotated in NR	48,085	57.13
Annotated in NT	36,210	43.02
Annotated in Swiss-Prot	33,036	39.25
Annotated in KEGG	30,353	36.06
Annotated in COG	20,941	24.88
Annotated in GO	33,527	39.84
Total unigenes	84,163	100
