# Supplementary material for: Transcriptomic Analysis of Green Leaf Plants and White–Green Leaf Mutants in Haworthia cooperi var. pilifera
Source: Genes (Basel). 2024 May 10;15(5):608. doi: 10.3390/genes15050608 (PMC11121492; doi:10.3390/genes15050608)
Supplement: Supplementary file 1 [file genes-15-00608-s001.zip › Table S3.pdf]

Table S3 DEGs of the two groups

Compare	Up	Down	All
LV, HUA	590	1,996	2,586
