# Supplementary material for: Transcriptomic Analysis of Green Leaf Plants and White–Green Leaf Mutants in Haworthia cooperi var. pilifera
Source: Genes (Basel). 2024 May 10;15(5):608. doi: 10.3390/genes15050608 (PMC11121492; doi:10.3390/genes15050608)
Supplement: Supplementary file 1 [file genes-15-00608-s001.zip › Table S4.pdf]

Table S4 KEGG annotation of DEGs (P value≤ 0.01)

Pathway	Number	P value	Q value	Pathway ID
Metabolic pathways	420	7.14E-07	1.83E-05	ko01100
Plant-pathogen interaction	149	6.31E-22	6.88E-20	ko04626
Glycerophospholipid metabolism	127	1.21E-05	1.38E-04	ko00564
Endocytosis	125	4.50E-05	3.77E-04	ko04144
Plant hormone signal transduction	124	2.00E-15	1.09E-13	ko04075
Ether lipid metabolism	117	1.89E-06	3.43E-05	ko00565
Starch and sucrose metabolism	68	0.000651595	4.18E-03	ko00500
Pentose and glucuronate interconversions	46	0.000109492	7.96E-04	ko00040
Phenylpropanoid biosynthesis	28	0.003058983	1.52E-02	ko00940
Stilbenoid, diarylheptanoid and gingerol biosynthesis	27	1.26E-05	1.38E-04	ko00945
Carotenoid biosynthesis	23	1.84E-05	1.82E-04	ko00906
Galactose metabolism	23	0.00010578	7.96E-04	ko00052
Nitrogen metabolism	22	4.95E-08	1.80E-06	ko00910
Other glycan degradation	22	8.35E-06	1.14E-04	ko00511
Flavone and flavonol biosynthesis	17	8.39E-07	1.83E-05	ko00944
Ascorbate and aldarate metabolism	17	0.000296948	2.02E-03	ko00053
Flavonoid biosynthesis	17	0.001840759	1.00E-02	ko00941
Limonene and pinene degradation	16	0.006129359	2.90E-02	ko00903
Glycosphingolipid biosynthesis - ganglio series	13	3.98E-06	6.20E-05	ko00604
Diterpenoid biosynthesis	13	4.43E-05	3.77E-04	ko00904

Glycosaminoglycan degradation	13	0.000845821	5.12E-03	ko00531
Sphingolipid metabolism	13	0.001178432	6.76E-03	ko00600
Biosynthesis of unsaturated fatty acids	11	0.009055237	4.11E-02	ko01040
Linoleic acid metabolism	8	0.002638935	1.37E-02	ko00591
