# Supplementary material for: Transcriptomic Analysis of Green Leaf Plants and White–Green Leaf Mutants in Haworthia cooperi var. pilifera
Source: Genes (Basel). 2024 May 10;15(5):608. doi: 10.3390/genes15050608 (PMC11121492; doi:10.3390/genes15050608)
Supplement: Supplementary file 1 [file genes-15-00608-s001.zip › Table S5.pdf]

Table S5 The differentially expressed genes related to plant pigment in leaf color mutant compared to control plant of *Haworthia cooperi* var. *pilifera*

ınction	Gene ID	log2 Ratio	up-down regulation	P value	FRD	Gene description
hloroplast vision	Unigene5427_All	-2.40870008	down	9.13E-07	0.000142	tubulin β-7 chain
	CL2099.Contig19_All	-2.16526084	down	1.22E-06	0.0001814	tubulin β-1 chain-like isoform 1
	CL2099.Contig8_All	-1.8927825	down	1.36E-05	0.0013319	Tubulin β-8 chain
	CL2099.Contig6_All	-1.56773889	down	0.00016369	0.0094047	Tubulin β-4 chain
	Unigene8230_All	-1.77373168	down	0.0005282	0.0224964	β-tubulin 2
	CL2099.Contig4_All	-2.18043304	down	0.00053477	0.0226687	tubulin β-1 chain-like isoform 1
	Unigene1981_All	2.018348859	up	1.45E-05	0.0014003	α-tubulin suppressor
ant pigment nthesis	CL11028.Contig1_All	-3.10557453	down	1.63E-12	1.84E-09	magnesium chelatase

CL5604.Contig1_All	-1.43372097	down	0.00095443	0.0348351	Probable chlorophyll(ide) b reductase NYC1
CL4410.Contig5_All	2.092666247	up	2.18E-07	4.31E-05	ζ-carotene desaturase
Unigene17993_All	1.317418677	up	0.00108391	0.0382206	ζ-carotene isomerase
CL4410.Contig4_All	1.390519217	up	0.00124997	0.042713	ζ-carotene desaturase
CL1839.Contig3_All	-1.50627327	down	0.000356	0.0168495	ζ-carotene desaturase
Unigene7511_All	-1.75886672	down	0.00042667	0.0192425	ζ-carotene desaturase
CL1839.Contig5_All	-1.57263871	down	0.00059144	0.0244243	ζ-carotene desaturase
Unigene7508_All	-1.78283725	down	0.00080739	0.0309188	ζ-carotene desaturase
CL504.Contig5_All	-2.46716559	down	3.43E-08	8.71E-06	Zeaxanthin epoxidase
Unigene11027_All	1.314412055	up	0.00107066	0.0379558	Zeaxanthin epoxidase
CL504.Contig2_All	-1.80965596	down	0.00115262	0.040083	Zeaxanthin epoxidase
Unigene4973_All	1.438920132	up	0.00034926	0.0166441	15-cis-ζ-carotene isomerase
CL13577.Contig7_All	-2.48171492	down	8.58E-09	2.62E-06	Predicted naringenin-chalcone synthase

CL13577.Contig3_All	-2.24188456	down	6.09E-05	0.0043906	Predicted naringenin-chalcone synthase
Unigene26705_All	-10.1949701	down	8.78E-05	0.005766	Predicted naringenin-chalcone synthase
Unigene26706_All	-1.61556237	down	0.00013207	0.0079331	Predicted naringenin-chalcone synthase
CL13577.Contig4_All	-2.20983127	down	0.00022635	0.0119495	Predicted naringenin-chalcone synthase
CL3495.Contig2_All	-3.048023982	down	3.95E-12	3.97E-09	flavonoid 3'-monooxygenase-like
Unigene632_All	-2.090751384	down	8.16E-06	0.000891337	flavonoid 3'-monooxygenase
Unigene20726_All	-10.15789874	down	1.21E-05	0.001215875	flavonoid 3'-monooxygenase
CL3495.Contig1_All	-3.530357842	down	0.000222926	0.011821191	flavonoid 3'-monooxygenase
CL6831.Contig1_All	-14.7153015	down	2.18E-09	8.42E-07	cytochrome P450
Unigene11481_All	-2.56523642	down	3.00E-09	1.11E-06	cytochrome P450 81D1-like
Unigene20404_All	-2.81708382	down	5.09E-06	0.0006094	Cytochrome P450 90D2
CL2201.Contig3_All	-1.8829742	down	7.49E-06	0.0008337	Cytochrome P450
Unigene17451_All	-4.50826667	down	9.91E-06	0.0010404	Cytochrome P450 94A1

CL1249.Contig6_All	3.187361737	up	0.00024554	0.012758	Cytochrome P450
CL4347.Contig1_All	-1.73764874	down	0.00030511	0.0150143	cytochrome P450 71B34-like
Unigene27172_All	1.487081466	up	0.00054274	0.02289	Cytochrome P450 71D8
Unigene42234_All	-5.01983932	down	0.00060952	0.0249725	cytochrome P450 94A1-like
Unigene11038_All	-1.32405972	down	0.00082282	0.0312796	cytochrome P450 allene oxide synthase
CL2201.Contig6_All	-1.67860822	down	0.00094467	0.0346156	cytochrome P450
Unigene4806_All	-3.3313211	down	0.0010471	0.0372697	Cytochrome P450 71A4
CL13369.Contig2_All	-1.59649585	down	0.00104818	0.0372924	cytochrome P450 81D1-like
CL4347.Contig2_All	-1.52984948	down	0.00119994	0.0413436	cytochrome P450 71B34-like
Unigene18224_All	5.648674207	up	0.0015035	0.0489332	Cytochrome P450 71A1
Unigene5170_All	-1.88410996	down	7.69E-05	0.0052257	Chlorophyll a-b binding protein 13
Unigene3942_All	-1.58495614	down	0.00018691	0.0104014	Photosystem II CP43 chlorophyll apoprotein
Unigene4090_All	-1.93294138	down	0.00019497	0.0107491	chloroplast chlorophyll a/b-binding protein

	Unigene4364_All	-1.37877774	down	0.00056958	0.0236388	light-harvesting chlorophyll a/b protein precursor
	Unigene3687_All	-1.37728013	down	0.00059884	0.0246808	chloroplast chlorophyll a/b-binding protein
	Unigene4242_All	-1.42572239	down	0.00088158	0.0328384	major chlorophyll a/b binding protein
anthocyanin ansport	CL6060.Contig3_All	-4.3410449	down	3.02E-18	1.57E-14	ABC-type Fe3+ transport system
	Unigene17867_All	-3.71303095	down	2.24E-11	1.77E-08	ABC transporter C family member 10-like
	CL3894.Contig4_All	-4.31091933	down	3.18E-09	1.16E-06	ABC transporter C family member 10-like
	CL2478.Contig4_All	-2.50069537	down	7.89E-09	2.49E-06	PDR5-like ABC transporter
	CL2478.Contig3_All	-2.45882435	down	1.90E-08	5.35E-06	ABC-type multidrug transport system, ATPase component
	Unigene26311_All	-2.94651659	down	8.09E-08	1.85E-05	ABC transporter C family member 10-like
	Unigene22921_All	-3.58237244	down	1.59E-07	3.34E-05	ABC transporter C family member 10
	CL5761.Contig2_All	-3.55635107	down	4.11E-07	7.42E-05	ABC transporter C family member 9
	CL5505.Contig4_All	-2.07357126	down	1.08E-06	0.000163	ABC-type uncharacterized transport system, permease component

Unigene42386_All	-4.4267268	down	3.18E-06	0.0004025	ABC transporter C family member 10-like
Unigene21844_All	-3.3151392	down	7.39E-06	0.0008253	ABC transporter C family member 10
Unigene42160_All	-13.5786214	down	9.42E-06	0.0009975	ABC transporter C family member 10-like
Unigene10228_All	-3.55925979	down	2.00E-05	0.0018041	ABC transporter C family member 10
CL2478.Contig2_All	-2.65825216	down	0.00010766	0.0067644	ABC-type multidrug transport system, permease component
CL5908.Contig1_All	1.588947592	up	0.00018687	0.0104014	ABC transporter B family member 28
Unigene14496_All	2.922295176	up	0.00024862	0.0128643	ABC-type multidrug transport system, ATPase component
Unigene16742_All	-1.48192665	down	0.00029792	0.0148075	ABC transporter
CL8468.Contig4_All	-1.53359781	down	0.00032657	0.0157887	ABC transporter D family member 1
CL8468.Contig6_All	-1.45801597	down	0.00050068	0.0215638	ABC transporter D family member 1
CL1076.Contig2_All	3.78675128	up	0.0006299	0.0256748	ABC transporter B family member 2
Unigene14524_All	-1.46672023	down	0.00083655	0.0316858	ABC-type multidrug transport system, ATPase component
CL9731.Contig1_All	1.705378176	up	0.00146444	0.0480754	ABC transporter B family member 28-like

CL342.Contig2_All	-5.04436083	down	2.78E-12	2.93E-09	MATE efflux family protein 1
CL12515.Contig1_All	-4.72883937	down	3.33E-07	6.17E-05	MATE efflux family protein 1
Unigene17547_All	-1.58658505	down	0.00081373	0.0310903	MATE efflux family protein 5
CL4078.Contig1_All	-1.38969374	down	0.0012163	0.0418087	MATE efflux family protein 5
CL4078.Contig2_All	-4.0306714	down	0.00142105	0.0469085	MATE efflux family protein 9
Unigene24280_All	-3.283938083	down	1.85E-09	7.46E-07	glutathione S-transferase
CL9650.Contig1_All	-2.630148405	down	4.11E-08	1.02E-05	glutathione S-transferase
CL1915.Contig1_All	-4.206256609	down	1.65E-07	3.42E-05	glutathione S-transferase
CL1915.Contig2_All	-4.663436737	down	1.52E-06	0.000217959	glutathione S-transferase
Unigene13934_All	-2.671397556	down	8.44E-06	0.000916897	glutathione S-transferase GSTU6
Unigene13933_All	-1.575520382	down	0.000185666	0.010365827	glutathione S-transferase U17 isoform 1
CL9349.Contig1_All	-1.97080243	down	0.000488245	0.021159737	Glutathione S-transferase T3
CL4943.Contig2_All	-2.45792307	down	0.000847201	0.032003343	glutathione S-transferase U17 isoform 1

transcription<br>ctor	CL13788.Contig2_All	-3.92006426	down	2.78E-16	9.26E-13	Triticum aestivum clone TaMYB18 R2R3-MYB protein mRNA
	CL13788.Contig1_All	-4.35646528	down	5.59E-12	5.23E-09	Myb superfamily proteins
	CL12514.Contig2_All	-2.33352498	down	3.11E-06	0.0003963	transcription factor MYB39
	Unigene17551_All	-2.1455495	down	3.28E-05	0.002674	MYB-like transcription factor DIVARICATA
	CL9836.Contig2_All	-2.19520296	down	9.24E-05	0.0059978	Myb family transcription factor APL
	CL11604.Contig2_All	-1.70590779	down	0.00010222	0.006462	Triticum aestivum clone TaMYB67 MYB-related protein mRNA
	Unigene28030_All	-2.68894741	down	0.00024107	0.0125749	myb-related transcription factor
	CL7266.Contig1_All	-2.64234443	down	0.00030323	0.0149908	Triticum aestivum clone TaMYB29 R2R3-MYB protein mRNA
	CL12936.Contig1_All	1.305921786	up	0.00094599	0.0346486	myb DNA-binding domain-containing protein
	CL13280.Contig2_All	-2.20547801	down	2.76E-06	0.0003593	MYB44_ARATH
	CL7266.Contig2_All	-2.96274799	down	4.87E-05	0.0036544	MYB39_ARATH
	CL2592.Contig5_All	-2.01586508	down	0.00010491	0.0066168	Myb family transcription factor APL

CL2592.Contig3_All	-1.96619295	down	0.00116127	0.0403167	Myb family transcription factor APL
CL846.Contig8_All	-2.14107322	down	1.32E-06	0.0001944	Vitis vinifera transcription factor bHLH118-like
CL6353.Contig1_All	-2.50929866	down	2.50E-06	0.0003322	Vitis vinifera transcription factor bHLH87-like
CL846.Contig4_All	-2.38266436	down	3.26E-06	0.0004094	putative HLH DNA-binding domain superfamily protein
CL846.Contig9_All	-2.1976101	down	7.29E-06	0.0008159	Transcription factor bHLH36
CL846.Contig2_All	-2.19806238	down	3.80E-05	0.0030172	putative HLH DNA-binding domain superfamily protein
CL5677.Contig2_All	-3.03002896	down	0.00015685	0.0091191	transcription factor bHLH92-like
Unigene7298_All	-1.64092089	down	0.00056431	0.0234785	Vitis vinifera transcription factor bHLH118-like
CL846.Contig5_All	-1.69272546	down	0.00069398	0.027777	Transcription factor bHLH36
CL3874.Contig2_All	-1.72550604	down	0.00086692	0.0324668	transcription factor bHLH150-like
Unigene14743_All	-1.43340106	down	0.00140072	0.0465154	Vitis vinifera transcription factor bHLH61-like
CL11133.Contig2_All	-4.34844495	down	1.79E-05	0.0016366	WD-40 repeat family protein
CL11133.Contig1_All	-2.78486288	down	2.63E-05	0.0022474	F-box and wd40 domain protein

CL4105.Contig1_All	-1.73845394	down	3.49E-05	0.0028208	WD-40 repeat family protein
CL6688.Contig4_All	1.41440984	up	0.00117354	0.040641	WD40 repeat
CL2610.Contig2_All	-1.79096934	down	5.36E-05	0.003969	WD40 repeat
CL3266.Contig5_All	-1.57359253	down	0.00039357	0.0181729	WD40 repeat
